# Nurse–Physician Collaboration and the Professional Autonomy of Intensive Care Units Nurses

**DOI:** 10.5005/jp-journals-10071-23149

**Published:** 2019-04

**Authors:** Delshad Aghamohammadi, Behrouz Dadkhah, Masoumeh Aghamohammadi

**Affiliations:** 1-3 Department of Nursing and Midwifery, Ardabil University of Medical Sciences, Ardabil, Iran

**Keywords:** Autonomy, Collaboration, Critical care unit, Nurse, Physician

## Abstract

**Background and aims:**

Poor collaboration between the physicians and the nurses may interfere with nursing performance in patient care. This study aimed to determine the nurse–physician collaboration and professional autonomy of intensive care nurses.

**Subjects and methods:**

This descriptive correlational study was performed on 126 nurses working in the intensive care units (ICUs) of Ardabil, Iran. The data were collected using the Jefferson scale of attitudes toward physician–nurse collaboration’ (JSAPNC) and the Dempster Practice Behavior Scale (DPBS). The results were analyzed using descriptive statistics (mean, standard deviation, and frequency) and inferential statistics (t-test, ANOVA, and Pearson).

**Results:**

The mean score of the nurse–physician collaboration was found to be 47.83 ± 3.9, which indicates good collaboration between physicians and nurses in the ICUs. The results showed that 73% of the nurses reported a moderate autonomy and 27% of them considered their autonomy to be high. There was no significant relationship between the nurse–physician collaboration and the professional autonomy of the nurses (*p* >0.05).

**Conclusion:**

The nurses who participated in this study had a positive attitude toward collaboration with the physicians and a moderate level of professional autonomy. Interventions may be required to further enhance the level of nurse–physician collaboration and the professional autonomy of nurses.

**How to cite this article:**

Aghamohammadi D, Dadkhah B, et al. Nurse-Physician Collaboration and the Professional Autonomy of Intensive Care Units Nurses. Indian J Crit Care Med 2019;23(4):178-181.

## INTRODUCTION

Teamwork is crucial for providing safe and high-quality care.^1^ Due to the complexity of care for critically ill patients, a teamwork attitude is essential.^2^ Collaboration is the process of interacting views between two or more people about a common theme.^3^ The goal of interprofessional collaboration is to create an equal opportunity for each team member so that they can share their knowledge and expertise in an environment filled with mutual trust and respect.^4^ The purpose of such collaboration in the healthcare system is that various healthcare professionals interact in patient care.^2^ Meanwhile, the concept of nurse–physician collaboration goes beyond just working together in a common environment. It requires a shared goal, reciprocal duty to provide high-quality care to resolve patient problems.^5^ Establishing effective professional collaboration between nurses and physicians is necessary because of their key roles in patient care and treatment.^6^ Evidence suggests that the nurse–physician collaboration is a major factor in improving disease outcomes including the mortality rate, readmission, and complications of the disease, as well as ventilator-associated pneumonia and bedsores.^7–9^ Furthermore, the lack of an appropriate professional relationship between the physicians and the nurses can lead to burnout^2^ and stress in nurses.^3^ Karanikola et al. showed that a low level of collaboration between the physicians and the nurses is an important factor in increasing the ethical stress of nurses.^10^ In real interprofessional collaboration, the two parties must be able to make independent decisions and have the power to implement their decisions.^2^ For good nurse–physician collaboration, it is necessary for both physicians and nurses to recognize their professional boundaries while maintaining a proper relationship. Each of them also needs to be involved in the decision making process based on their expertise to achieve patient's healthcare outcomes.^11^ The specialized duties of nurses and physicians require autonomy.^12^ Since autonomy is the cornerstone of any profession, nurses seek to strengthen their professional level by promoting clinical autonomy.^13^ The professional autonomy of nurses is defined as the ability to have the right and the responsibility for doing their job to decide on the patient's needs and the freedom of action.^2^ From Dorgham's point of view, an important factor in professional autonomy of nurses is their decision-making ability, which leads to the establishing the basis of professional knowledge.^14^ A study on ICU nurses in Greece reported moderate levels of autonomy in technical tasks and low autonomy levels in the decision making process.^15^ The results of the study conducted by Dorgham and Al-Mahmoud also showed a low level of professional autonomy in Egyptian nurses.^14^ Low autonomy is one of the most important reasons for nurses wanting to transfer to nonclinical units.^16^ Moreover, the ability of nurses to independently perform nursing services plays an essential role in nursing job satisfaction.^13^

**Table 1:** The mean, standard deviation, and frequency of attitude scores of participants on the nurse–physician collaboration

*Variable*	*Mean*	*Standard deviation*	*Min*	*Max*	*Frequency percentage of collaboration*		
					*Good*	*Moderate*	*Weak*
Nurse–physician collaboration (15–60)	47.83	3.9	32	58	71.4	28.6	0
Joint education and collaboration field (7–28)	23.21	2.26	14	28	78.6	20.6	0.8
Care field (3–12)	9.89	1.08	6	12	67.5	31.7	0.8
Nurses’ autonomy field (3–12)	9.91	1.29	6	12	54	45.2	0.8
Authority or domination of physician field (2–8)	4.8	1.23	2	8	6.3	54.8	38.9

The presented definitions for interprofessional collaboration and professional autonomy suggest that both require a decision making power as well as the ability to apply those decisions and to take responsibility for them. Therefore, it seems that interprofessional collaboration and professional autonomy are interrelated; these influence the quality of care and patient outcomes, care costs, rate of resignation, and the physical and mental health of nurses.^17^ Today, in modern healthcare systems, one of the major goals is to change the attitude from superiority of treatment to providing care.^18^ An essential element for offering high-quality care is the effective collaboration between the physician and the nurse, which ensures the exchange of information and hence strengthens the therapy.^3^ On the contrary, if the nurses’ professional autonomy is achieved, the patients’ care needs are carefully identified by the nurses, and the necessary nursing measures are taken.^17^ Considering the above-mentioned issues, as well as the lack of studies on the relationship between the nurse–physician collaboration and the professional autonomy in the nurses of Iran, especially in the ICUs, this study aimed to determine the status of collaboration between the physicians and the nurses and the professional autonomy of the nurses in the ICUs of the educational centers of Ardabil, Iran.

## MATERIALS AND METHODS

This was a descriptive correlational study. The research population consisted of all 150 nurses working in the ICUs of the three Educational Centers in Ardabil. Among them, 126 full-time nurses with a bachelor's degree and above, who were interested in participating in the study, were enrolled using a census method. Data were collected using the JSAPNC and the DPBS. The Jefferson scale includes 15 statements, which were collected under four subscales, i.e., shared education and teamwork (seven statements), caring versus curing (three statements), nurses’ autonomy (three statements), and physicians’ authority (two statements). The Jefferson Scale total score ranges from 15 to 60, with higher values indicating more positive attitude toward physician–nurse collaborative relationships.

The DPBS is a 30-item instrument developed with a Likert-type format and a 5-point scaling that focuses on overt and covert behaviors, actions, and conduct related to the extent of an individual's autonomy in a practice setting. The content validity index (CVI) was used to determine the content validity of the questionnaires. For this purpose, the questionnaires were submitted to 11 nursing faculty members of the Ardabil University of Medical Sciences and examined for relevance, simplicity, and clarity of statements. The Jefferson scale with a CVI of 0.87 and DPBS with a CVI of 0.95 was confirmed. Cranach's α-coefficients of 0.78 and 0.83 indicated, respectively, the reliability of the collaboration and the professional autonomy scales. The data were analyzed using descriptive statistics (frequency, mean, and standard deviation) and inferential statistics (t-test, ANOVA, and Pearson correlation) in SPSS ver. 21.

## RESULTS

### Demographics

The results showed that among 126 critical care units nurses, women and men accounted for 125 (99.2%) and one (0.8%) of the subjects, respectively. Most participants (60.3%) were in the age range of 30–40 years. Most of the samples (67.5%) were also married and had a bachelor's degree in nursing (96.8%), and many had less than 5 years of experience in the critical care units (38.9%).

### Nurse–Physician Collaboration

Regarding the nurse–physician collaboration, the results showed that this cooperation was assessed to be at an appropriate level by the majority of the nurses (71.4%). Among the nurse–physician collaboration fields, the highest average score was obtained in the field of joint education and collaboration (23.21 ± 2.26), and the lowest score related to the authority or domination of physicians (4.8 ± 1.23). The statements of “The nurse should be considered as a physician's colleague, not a physician's assistant” and “physicians should be responsible and competent at all healthcare topics” received the highest and lowest scores, respectively (Table 1).

### Professional Autonomy

Regarding the professional autonomy of the critical care unit nurses, the results showed that 73% and 27% of the nurses considered their independence to be moderate and high, respectively; and among the areas of professional autonomy, the highest and lowest score was obtained in the realization (35.38 ± 4.03) and readiness fields (35.13 ± 6.4) (Table 2).

### Nurse–Physician Collaboration and Professional Autonomy

Concerning the relationship between the critical care units nurses’ attitudes toward nurse–physician collaboration and professional autonomy, the results of the Pearson correlation test showed no significant relationship between the variables above (*p* = 0.023, *r* = 0.114) (Table 3).

## DISCUSSION

The results of this study showed that 71.4% of the critical care units’ nurses had a positive attitude toward nurse–physician collaboration. Also, the highest and lowest scores related to the fields of “joint education and collaboration” and “authority or domination of physicians”, respectively. In a study on the nurses of Johannesburg ICUs, Le Roux and colleagues concluded that the nurses had a positive attitude toward nurse–physician collaboration.^19^ Several other studies that were conducted using JSAPNC scale reported that the nurses had good attitudes toward their collaboration with physicians.^20–24^ However, Giorgio et al. in a study in Cyprus showed that the nurses’ attitude toward collaborating with physicians was at a moderate level.^2^ Cotter also studied the attitudes of nurses working in different wards of the hospital about the nurse–physician collaboration, using the nurse–physician collaboration scale (NPCS) and assessed it at a moderate level.^25^ It seems that the nurses’ workplace is one of the influential factors of the nurses’ attitude toward collaboration with the physicians; so that in the ICUs where the interaction between colleagues is high, the nurses’ attitude toward collaboration with the physicians is reported to be desirable.^3^

**Table 2:** Mean, standard deviation and frequency of attitude scores of participants on professional autonomy

*Variable*	*Mean*	*Standard deviation*	*Min*	*Max*	*Frequency percentage of professional autonomy*		
					*Good*	*Moderate*	*Weak*
Professional autonomy (30–150)	104.84	11.51	74	138	27	73	0
Readiness field (11–55)	35.13	6.4	18	49	23	72.2	4.5
Empowerment field (7–35)	23.99	3.13	16	32	17.5	81.7	0.8
Realization field (9–45)	35.38	4.03	26	45	74.6	25.4	0
Assessment field (3–15)	10.33	2.39	5	15	35.7	52.4	11.9

**Table 3:** Relationship between nurse–physician collaboration and nurses’ professional autonomy working in critical care units of Ardabil's educational centers

*Variables*	*Nurse–Physician collaboration*					
	*Number*	*Total collaboration*	*Joint education and collaboration field*	*Care field*	*Nurses' autonomy field*	*Physicians' domination field*
Total autonomy	126	r = 0/114 p = 0/203	r = 0/05 p = 0/54	r = 0/09 p = 0/31	r = 0/29 p = 0/001	r = –0/128 p = 0/15
Readiness field	126	r = 0.05 p = 0.56				
Empowerment field	126	r = 0.12 p = 0.18				
Realization field	126	r = 0.16 p = 0.06				
Assessment field	126	r = –0.02 p = 0.78				

About the status of the professional autonomy of the critical care units’ nurses, the results showed that most nurses assessed their professional autonomy at a moderate level 104.84 ± 11.51). Cotter also reported that the professional autonomy score of the Irish emergency nurses was 104.54 ± 12.53, which is consistent with the present study.^25^ Kramer and Schmalenberg confirmed that little changes have been made over the past 20 years in nurses autonomy.^26^ Other studies reported higher levels of professional autonomy in nurses.^27,28^ However, Amini et al. showed that the mean and standard deviation of the professional autonomy score of the nurses was 90.7 ± 13.3.^17^ Comparing the results of the various studies shows that most of the studies in which the nurses’ autonomy scores have been reported to be high relate to the United States, where the nurses have a higher authority. In addition, the difference between the nurses’ level of professional autonomy can be due to the hierarchical relationship between the physicians and the nurses, and the high work load on the healthcare systems in Iran.^29,30^ We found that there was no significant relationship between the nurses’ attitudes toward the nurse–physician collaboration and the professional autonomy of nurses. Gagnon et al. showed that nurse–physician collaboration has a positive impact on the clinical autonomy of nurses,^31^ but Stewart and colleagues considered collaboration as an obstacle to the professional autonomy of the nurses.^32^ Maylon et al. also found no significant relationship between the professional autonomy of nurses and the collaboration between the physicians and the nurses, this finding is consistent with the results of the present study.^28^ Since this study was conducted only in ICUs, the results are not generalized to other wards. In addition, the self-report nature of the questionnaire and the complexity of the concepts of nursing collaboration^33^ and autonomy^34^ should also be considered.

## CONCLUSION

The findings of this study indicated a high level of collaboration between the physicians and the nurses and a moderate level of professional autonomy among the critical care units’ nurses in Ardabil, Iran. Given the lack of a statistically significant relationship between the two concepts in this study, there is a need to clarify the above concepts further. Also, in order to improve the autonomy status of the nurses, their participation in clinical decision-making should be one of the goals of each organization. In addition, educational interventions to strengthen the critical thinking approach^33^ can help to improve the nursing professional autonomy. Furthermore, due to the importance and necessity of collaboration between the physicians and the nurses in improving the outcomes of the patient,^35,36^ it is necessary to use different approaches, such as holding workshops on ways to improve communication and the use of team rounds in medical centers.
